# Plasma proteomic signature of the risk of developing mobility disability: A 9‐year follow‐up

**DOI:** 10.1111/acel.13132

**Published:** 2020-03-10

**Authors:** Yusuke Osawa, Richard D. Semba, Giovanna Fantoni, Julián Candia, Angélique Biancotto, Toshiko Tanaka, Stefania Bandinelli, Luigi Ferrucci

**Affiliations:** ^1^ Longitudinal Study Section Translational Gerontology Branch National Institute on Aging National Institutes of Health Baltimore MD USA; ^2^ Wilmer Eye Institute Johns Hopkins University School of Medicine Baltimore MD USA; ^3^ Clinical Research Core National Institute on Aging National Institutes of Health Baltimore MD USA; ^4^ Laboratory of Human Carcinogenesis Center for Cancer Research National Cancer Institute NIH Bethesda MD USA; ^5^ Precision Immunology, Immunology and Inflammation Research Therapeutic Area Sanofi Cambridge MA USA; ^6^ Geriatric Unit Azienda Sanitaria di Firenze Florence Italy

**Keywords:** cathepsin S, growth/differentiation factor 15, mobility disability, proteomics, thrombospondin‐2

## Abstract

**Introduction:**

Mobility disability is a powerful indicator of poor health in older adults. The biological and pathophysiological mechanism underlying the development of mobility disability remains unknown. This study conducted a data‐driven discovery phase investigation to identify plasma proteins that predict the incidence of mobility disability in community‐dwelling older adults without mobility disability at baseline.

**Methods:**

We investigated 660 women and men, aged 71.9 ± 6.0 (60–94) years, who participated in the Invecchiare in Chianti, “Aging in the Chianti Area” study and completed the 400‐m walk at fast pace (400‐m walk) at enrollment. Median follow‐up time was 8.57 [interquartile, 3.20–9.08] years. SOMAscan technology was used to measure 1,301 plasma proteins at enrollment. The incident of mobility disability was defined as inability to complete the 400‐m walk. Protein‐specific Cox proportional hazard model was adjusted for sex, age, and other important covariates.

**Results:**

Plasma levels of 75 proteins predicted mobility disability (*p* < .05). Significant proteins were enriched for the KEGG “PI3K‐Akt signaling,” “phagosomes,” and “cytokine–cytokine receptor interaction” pathways. After multiple comparison adjustment, plasma cathepsin S (CTSS; hazard ratio [HR] 1.33, 95% CI: 1.17, 1.51, *q* = 0.007), growth/differentiation factor 15 (GDF15; HR: 1.45, 95% CI: 1.23, 1.72, *q* = 0.007), and thrombospondin‐2 (THBS2; HR: 1.44, 95% CI: 1.22, 1.69, *q* = 0.007) remained significantly associated with high risk of losing mobility.

**Conclusion:**

CTSS, GDF15, and THBS2 are novel blood biomarkers associated with new mobility disability in community‐dwelling individuals. Overall, our analysis suggests that cellular senescence and inflammation should be targeted for prevention of mobility disability.

## Graphical abstract

Among 1,301 plasma proteins assessed, cathepsin S, thrombospondin‐2, and growth/differentiation factor 15 significantly and independently predicted mobility loss in 660 community‐dwelling adults. Proteins associated with mobility loss were enriched for senescence‐associated secretory phenotype and for proteins in the PI3K‐Akt, phagosome, or cytokine–cytokine receptor interaction pathways. High plasma levels of SASP‐induced proteins related to inflammation and phagocytes activation mark a condition of high risk of mobility loss.

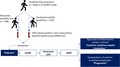

## 1 INTRODUCTION

The prevalence of functional limitations and disability increases with aging (Satariano et al., 2012). Mobility disability, often assessed as subjective or objective difficulties in gait performance, not only relates to poor health‐related outcomes such as mortality (Newman et al., 2006), but also strongly affects psychological health and social isolation (Satariano et al., 2012). Once mobility disability develops in older individuals, its reversibility is limited and short lasting (Leveille, Penninx, Melzer, Izmirlian, & Guralnik, 2000). Except for a limited beneficial effect of physical activity, no other effective interventions are currently available for the prevention of mobility disability in late life (Manini et al., 2017). Thus, it is a public health imperative to develop a better understanding of the biological mechanisms that cause loss of mobility in older age and to identify new intervention targets that effectively prevent and slow down the clinical course of mobility loss. The Targeting Aging with Metformin workgroup (Justice et al., 2018) recently identified a number of candidate biomarkers that could be used to track changes in health and functional status with aging such as interleukin 6, C‐reactive protein, and tumor necrosis factor‐α RII as biomarkers for inflammation and intercellular signaling, growth/differentiation factor 15 (GDF15) as a biomarker for chronological aging, cystatin C as a biomarker for kidney function, and NT‐proBNP as a biomarker for cardiovascular health. However, this approach is limited to the range of biomarkers that are already available and is based on incomplete understanding of the biological pathways involved in mobility loss with aging. An agnostic, discovery approach based on multiple biomarkers may be more appropriate for the identification of still poorly understood dysfunctional pathways involved in the disablement process. Ideally, these biomarkers should be studied in biological fluids that are easily accessible, making the findings immediately translatable to clinical applications.

Performing discovery proteomics in plasma and serum is extremely challenging because of the large number of proteins with wide concentration ranges and highly abundant proteins that interfere with the quantification of less abundant ones (Geyer et al., 2016). Recent advances in technology—such as aptamer‐based platforms that can measure thousands of proteins in small samples—have partially overcome these limitations and can be used to identify biological and pathophysiologic pathways involved in mobility loss with aging (Semba et al., 2019) and biological aging in general (Tanaka et al., 2018). Proteins are direct effectors of biological mechanisms and closer than any other “omics” to clinical phenotypes. In fact, protein biomarkers are already extensively used in clinical practice. However, little is known about the relationship of a comprehensive evaluation of plasma proteome with the development of mobility disability. The goal of this project was to conduct a hypothesis‐free, data‐driven discovery phase investigation to identify plasma proteins that predict incident mobility disability in community‐dwelling older adults free of mobility disability at baseline.

## 2 RESULTS

Among 1,453 men and women who enrolled in the Invecchiare in Chianti, “Aging in the Chianti Area” (InCHIANTI) study, conducted in Tuscany, Italy, we selected 660 women and men aged 60 and older who had plasma proteomics data and completed 400‐m walk test at fast pace at baseline visit and who had also concurrent data on body mass index (BMI), education level, smoking status, physical activity, global cognitive assessment, and depression symptoms at baseline visit. Table 1 shows baseline characteristics of the participants. Of the 660 participants studied at baseline, over the 9‐year follow‐up, 292 participants (44.2%) developed mobility disability, defined as inability to complete a 400‐m walk at fast pace, 368 had not developed mobility disability at their last follow‐up visit, 105 had died, and 38 were lost to follow‐up. Compared to those who were censored, participants who developed mobility disability were older, more likely to be female, less educated, higher BMI, less physically active, and with poorer cognition, greater multi‐comorbidity, and slower gait speed at baseline.

**Table 1:** Participant characteristics at baseline visit

	Overall (*n* = 660)	Censored^†^ (*n* = 368)	Developed mobility disability (*n* = 292)	*p*‐value
	Mean ± *SD*	Mean ± *SD*	Mean ± *SD*	
Age (years)	71.9 ± 6.0	70.3 ± 5.5	73.9 ± 6.1	**<.0001**
Sex (women, %)	53.9	48.6	60.6	**.004**
Education level (6 years or more, %)	30.8	38.9	20.6	**.001** *
Body mass index (kg.m^2^)	27.5 ± 3.9	27.2 ± 3.5	27.8 ± 4.2	**.01** *
Current smoker (%)	15.3	16.6	13.7	.70*
Inactive (%)	57.6	48.6	68.8	**.0001** *
MMSE (0–30)	26.0 ± 2.8	26.4 ± 2.5	25.4 ± 3.0	**.01** *
CES‐D total score (0–60)	11.7 ± 8.5	10.7 ± 8.0	12.9 ± 8.9	.09*
Gait speed in 400 m at fast pace (m/s)	1.2 ± 0.2	1.3 ± 0.2	1.1 ± 0.2	**<.0001** *

Abbreviations: CES‐D, Center for Epidemiologic Studies depression scale; MMSE, Mini‐Mental State Examination

^*^ Age‐ and sex‐adjusted *p*‐values.

^†^ For those who did not develop mobility disability, the follow‐up time was censored at the date of 9‐year follow‐up visit. We treated those who died during the follow‐up period without developing mobility disability as censored at the day of their death and those who missed follow‐up without developing mobility disability as censored at the last follow‐up visit day.

**Figure 1:**
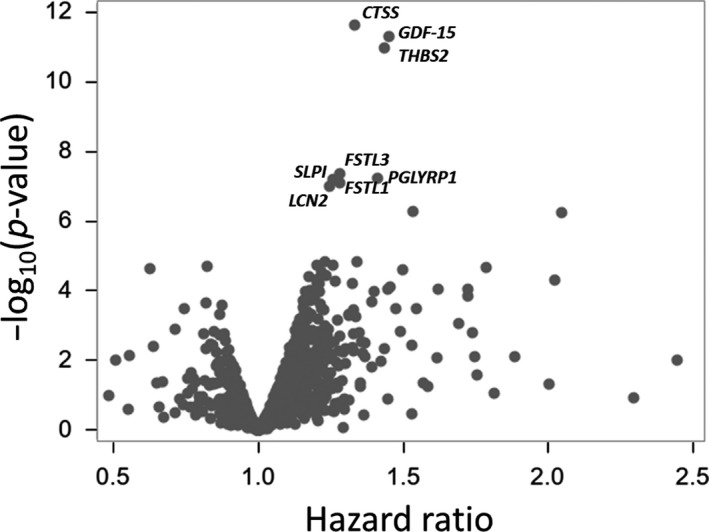
Association between plasma proteins and the risk of developing mobility disability. Volcano plot summarizing association of 1,301 proteins and risk of developing mobility disability. Hazard ratios and p‐values are from multivariate Cox proportional hazard models adjusted for age, sex, BMI, education year, smoking status, physical activity, MMSE, CES‐D, and baseline gait speed in 400‐m walk at fast pace

Table S1 shows a list of 1,301 proteins sorted by false discovery rate (FDR) *q*‐values and *p*‐values from protein‐specific Cox proportional hazard models adjusted for baseline covariates including age, sex, BMI, education level, smoking status, baseline physical activity, Mini‐Mental State Examination score (MMSE), depressive symptoms (the Center for Epidemiologic Studies Depression Scale, CED‐S), and baseline gait speed. Because proteins are measured in units of relative abundance, hazard ratios (HR) should be interpreted as the increased risk of developing mobility disability associated with 1‐unit standard deviation increase in plasma protein. Among 1,301 proteins, 75 proteins were associated with differential risk of developing mobility disability with a *p*‐value <.05. After adjusting for multiple comparisons, three proteins were still significantly associated with increased risk of developing mobility disability, namely cathepsin S (CTSS; HR: 1.33, 95% CI: 1.17, 1.51, FDR *q* = 0.007), a lysosomal cysteine that participates in the degradation of antigenic proteins to peptides for presentation on MHC class II molecules; growth/differentiation factor 15 (GDF15; HR: 1.45, 95% CI: 1.23, 1.72, FDR *q* = 0.017), a cytokine, distant member of the transforming growth factor (TGF)‐β superfamily; and thrombospondin 2 (THBS2; HR 1.44, 95% CI: 1.22, 1.69, FDR *q* = 0.007), a glycoprotein that mediates cell‐to‐cell and cell‐to‐matrix interactions involved in cell adhesion and migration (Figure 1, Table 2). When these three significant proteins were jointly introduced into a model predicting mobility loss, higher circulation levels in all three proteins were still significantly and independently associated with higher risk of mobility disability (Table S2). We did not find any significant interactions between sex and level of the three significant proteins in predicting new mobility disability (CTSS, *p* = .23; GDF15, *p* = .41; THBS2, *p* = .37).

**Table 2:** Significant plasma proteins for incident mobility disability in protein‐specific Cox proportional hazard risk model

Gene symbol	Entre GeneID	Target	Model				
			HR	95%CI		Raw	FDR
						*p*‐value	*q*‐value
CTSS	1520	Cathepsin S	1.33	1.17	1.51	<.0001	0.007
GDF15	7058	MIC−1	1.45	1.23	1.72	<.0001	0.007
THBS2	9518	TSP2	1.44	1.22	1.69	<.0001	0.007

Note

Model was adjusted for sex, baseline age, baseline BMI, education level, baseline smoking status, baseline physical activity, baseline MMSE, baseline CED‐S, and baseline gait speed.

Despite significant association with risk of mobility loss when nominal *p*‐values were considered, other 72 proteins were no longer associated with the risk of mobility disability in FDR adjustment (*q* < 0.05). However, to gather more information from our analysis we used two approaches. First, considering the strictness of FDR adjustment, we regarded proteins that had nominal p‐value less than 0.001 in a single protein model as promising proteins. This strategy included five proteins (follistatin‐like 1 [FSTL1]; peptidoglycan recognition protein 1 [PGRP‐S]; secretory leucoprotease inhibitor [SLPI]; follistatin‐like 3 [FSTL3]; and lipocalin 2 [LCN2]), in addition to the three most significant proteins described above. Second, because mobility disability is a complex trait, it is likely a multiple protein model will have greater predictability over a single protein model. In order to identify a multi‐protein model of mobility disability, the other approach was to include all proteins with a nominal *p*‐value <.05 (75 proteins) into a Cox regression model including demographic covariates and then perform a backward selection to identify protein that was independently associated with mobility loss. In this model, both demographic covariates and the three significant proteins identified above were forcefully retained in the model. In the final model, 17 proteins were retained as significant (ADP‐ribosyl cyclase/cyclic ADP‐ribose hydrolase 1 [CD38]; myostatin [MSTN]; stromelysin‐1 [MMP3]; basal cell adhesion molecule [BCAM]; contactin‐1 [CNTN1]; peptidoglycan recognition protein 1 [PGLYRP1]; N‐acetylglucosamine‐6‐sulfatase [GNS]; natriuretic peptides B [NPPB]; tyrosine‐protein kinase Fgr [FGR]; thyroid peroxidase [TPO]; spondin‐1 [SPON1]; sialoadhesin [SIGLEC1]; carbohydrate sulfotransferase 15 [CHST15]; collagenase 3 [MMP13]; mitogen‐activated protein kinase 13 [MAPK13]; tumor necrosis factor ligand superfamily member 14 [TNFSF14]; and ferritin heavy chain [FTH1]) (Table S3). Of note, many of the proteins in this list can be associated with pathways whose role in functional decline with aging has been previously hypothesized (Tanaka et al., 2018).

With the aim of identifying biological pathways involved in the process leading to mobility disability, we performed enrichment analyses on the 75 top proteins with nominal p‐value *p* < .05. Three pathways were enriched: “PI3K‐Akt signaling” (*p* = .048), a key pathway to regulate cell cycle, including cell survival, proliferation, and growth (genes, EPH receptor 2 [EPHA2], ephrin A2 [EFNA2], erythropoietin [EPO], growth hormone 1 [GH1], THBS2, and vascular endothelial growth factor D [VEGFD]); “phagosome” (*p* = .002), a pathway related to the function of macrophages (genes, Fc fragment of IgG receptor IIIb [FCGR3B], CTSS, macrophage scavenger receptor 1 [MSR1], myeloperoxidase [MPO], oxidized low‐density lipoprotein receptor 1 [ORL1], and THBS2); and “cytokine–cytokine receptor interaction” (*p* = .003), a pathway playing a key role in inflammatory processes (genes, C‐C motif chemokine ligand 14 [CCL14]; C‐C motif chemokine ligand 14 [CCL16]; erythropoietin [EPO]; GH1; interleukin 1 receptor type 1 [IL1R1]; leptin receptor [LEPR]; and TNFSF14). Of note, of the 75 nominally significant proteins, 20 have been previously identified as belonging to the senescence‐associated secretory phenotype by the SASP Atlas, including THBS2 and GDF15 (Basisty et al., 2019; Table S4).

## 3 DISCUSSION

We found that among 1,301 plasma proteins quantified, higher plasma CTSS, THBS2, and GDF‐15 significantly predicted greater risk of mobility disability in community‐dwelling adults. Prior to multiple comparison adjustment, we also found several other proteins that were significantly associated with mobility disability, and in KEGG pathway analysis, they were enriched for the “PI3K/Akt”, “phagosome”, and “cytokine–cytokine receptor interaction” pathways. To our knowledge, this is the first study that connected these three proteins and three pathways to the risk of developing disability in older adults. The robustness of these findings should be tested in other, possibly larger, longitudinal studies. Nevertheless, they point to specific biological mechanisms that deserve further investigation.

Cathepsin S (CTSS), a 32–42 kDa protein coded by *CTSS* on chromosome 1q21.3), is a member of the cysteine cathepsin protease family. CTSS is expressed in activated macrophages and advanced atherosclerotic lesions (Tjondrokoesoemo et al., 2016; Turk et al., 2012). A number of studies have shown that the cysteine cathepsin proteases are implicated in extracellular matrix remodeling, which is one of the main mechanisms of atherosclerotic development and instability (Lutgens, Cleutjens, Daemen, & Heeneman, 2007). A pathogenetic role of the cysteine cathepsin protease has been found in atherosclerosis, myocardial infarction, cardiac hypertrophy, cardiomyopathy, abdominal aortic aneurysms, and hypertension. In particular, the cysteine cathepsin protease regulates the adaptive and innate immune response via antigen presentation in antigen‐presenting cells (Liu et al., 2018). In addition, by activating Toll‐like receptor (TLR)7 and TLR9, CTSS expression suppresses the immune activity of regulatory T cells and this mechanism may contribute to a pro‐inflammatory state (Gocheva et al., 2010). A longitudinal study demonstrated that higher serum CTSS predicted higher risks of cardiovascular‐ and cancer‐specific mortalities (Jobs et al., 2011). Our study additionally suggested that CTSS could be an early marker of deterioration of the physiological systems that are involved in the maintenance of mobility, possibly by its effect on cardiovascular fitness required to complete the 400‐m walk (Simonsick et al., 2001).

Growth/differentiation factor 15 (GDF15) is a 16.7 kDa protein coded by *GDF15* on chromosome 19p13.11. GDF15, a cytokine and distant member of the transforming growth factor (TGF)‐β superfamily, is expressed in response to tissue injury such as cardiovascular injury (Xu et al., 2006). The pleiotropic biological role of GDF15 is still a matter of much debate, and there is evidence for both anti‐inflammatory and pro‐inflammatory roles such as in the context of atherosclerosis (Emmerson, Duffin, Chintharlapalli, & Wu, 2018). GDF15 is considered as a SASP and marker of systemic energy homeostasis (Justice et al., 2018). Epidemiological studies consistently demonstrated that the expression of GDF15 is up‐regulated with aging (Tanaka et al., 2018) and higher GDF15 relates to poor physical functions (Semba et al., 2019) and higher risk of diabetes, cancer, cognitive impairment, cardiovascular diseases, and mortality (Justice et al., 2018). It has been proposed that overexpression of GDF15 may represent “body's compensatory response” to the tissue injury (Emmerson et al., 2018). The present results further suggest the independent effects of these three proteins and the cumulative effects of upregulations in these proteins on the development of mobility disability.

Thrombospondin‐2 (THBS2) is a 150–160 kDa protein encoded by *THBS2* on chromosome 6q27. THBS2 belongs to a group of matricellular proteins that plays a fundamental role in maintaining the architectural continuity of heart and blood vessels (Chistiakov, Melnichenko, Myasoedova, Grechko, & Orekhov, 2017) as well as in cartilage differentiation and prevention of chondrocyte hypertrophy (Jeong et al., 2015). In advanced atherosclerotic lesions, THBS2 stimulates phagocytic function of macrophages, and this activity has been interpreted as anti‐atherogenic (Chistiakov et al., 2017). THBS2 gene is overexpressed in response to damage or during remodeling in heart failure, hypertensive cardiac disease, hypertrophic heart (Schroen et al., 2004), and atherosclerosis (Streit et al., 1999) and in the context of these diseases may act as an anti‐angiogenic function (Schroen et al., 2004). However, the overexpression of THBS2 is considered as an attempt to compensate for the ongoing damage in these pathological conditions although its effectiveness is limited (Chistiakov et al., 2017). An epidemiological study demonstrated that elevated expression of THBS2 gene predicted higher risk of mortality (Shi & Tian, 2019), suggesting high expression of THBS2 marks a condition of risk of health deterioration, although whether it is causally linked to health instability or is a resilience biomarker is not known. This view is consistent with our finding that higher plasma THBS2 is a higher risk factor for developing mobility disability.

Because of the high risk of finding false positive association in discovery studies that screen large number of variables, we focused mainly on proteins that were significant at the level of a conservative FDR. However, when all 75 proteins that were associated with the risk of developing mobility disability with a nominal *p* < .05 value were evaluated for enrichment of specific pathways, we found that “PI3K‐Akt,” “phagosome,” and “cytokine–cytokine receptor interaction” were significantly enriched. This finding suggests that in the pathway that eventually leads to mobility loss, atherosclerosis and cellular immunity play important roles. FSTL1 appears to protect against oxidized low‐density lipoprotein‐induced inflammation in endothelial cells, a process that is also important in atherogenesis (Guo, Liang, Li, & Long, 2016). While some key proteins identified in this study are expressed throughout atherogenesis, other proteins are only expressed in advanced atherosclerosis. Throughout the progression of atherosclerosis, PGLYRP1 is expressed in macrophages within atherosclerotic lesions, and its plasma level may reflect inflammation state due to chronic infection (Guo et al., 2016). TNFSF14 is also expressed in macrophages and is implicated in atherogenesis by inducing pro‐inflammatory cytokines and matrix metalloproteinases (Lee et al., 2001). Expression of MSTN in the media, neointima, plaque shoulder and infiltrating macrophages is implicated in the progression of atherosclerosis (Verzola et al., 2017). In the circulation, the biological activity of plasma myostatin can be inhibited by various binding proteins. In the present study, among myostatin antagonists, only FSTL3 was one of the top ranked proteins. LCN2, which is also expressed in macrophages within atherosclerosis lesions, is involved in atherogenesis by advancing polarization and migration of monocytic cells and development of foam cells (Oberoi et al., 2015). On the other hand, protective roles of SLPI have been demonstrated against progression of atherosclerosis (Zhong, Wang, Li, Peng, & Jiang, 2017). Pleiotropic roles of GDF15 have been implicated in both pro‐ and anti‐inflammation throughout the progression of atherosclerosis (Emmerson et al., 2018). While atherosclerosis is partly attributable to accumulated senescent cells and the SASP and could be a possible upstream of disability (Ferrucci & Fabbri, 2018), our proteins notably included various SASP members (Table S4). In our expanded multivariate analysis, we selected another set of proteins that were independently and significantly associated with higher risk of mobility loss among those that were nominally associated with mobility loss in pairwise analyses. This set included proteins that have been associated with the senescent pro‐inflammatory phenotype (MMP3, MMP13) (Roeb, 2018; Yan, 2015) and proteins biomarkers that have been associated with inflammation (NPPB, BCAM, TPO, CD38, FGR, SIGLEC1, CHST15, MAPK13; Amici et al., 2018; Munthe‐Fog et al., 2012; O'Neill, Berg, & Mullen, 2013; Tribulatti, Carabelli, Prato, & Campetella, 2019). These findings raise the idea that cellular senescence and inflammation play a role in the disablement process. This is consistent with data in the literature showing that the pro‐inflammatory state of aging plays a pivotal role in developing mobility disability (Ferrucci & Fabbri, 2018) and recent literature showing that the accumulation of P16‐positive cell in intramuscular adipose tissue is associated with poor lower extremity performance in older persons (Justice et al., 2018). Even though all participants were disability‐free at baseline, some may have already experienced a subclinical level of biological aging (Ferrucci, Levine, Kuo, & Simonsick, 2018). THBS2 and GDF15 overexpression status at baseline may represent an attempt to counterbalance the pro‐inflammatory state or damaged tissues and may play role in alleviating damaged tissues.

Our study has several strengths. An important strength is the longitudinal design with a highly representative population‐based samples and standardized protocols for assessing mobility function in addition to diversity in sex and with low attrition rate during the follow‐up. We explored a wide range of proteins and performed conservative analyses, highlighting only those proteins that were strongly associated with the risk of mobility disability after FDR correction. Our study also has limitations. First, in the aptamer‐based platform used in this study, circulating protein complexes with myostatin were not disrupted by acid dissociation or other means; if aptamers are blocked by complexes, this may interfere with measurement. SOMAscan provides relative but not absolute quantification. For this reason, it would be important to confirm these results in other independent population as well as using other assay methods for protein quantification. Second, while we assessed 1,301 proteins, this is not a comprehensive assessment of the plasma proteome. Future studies should confirm these results using other proteomic assessment, including an updated version of the SOMAscan that may allow for the measurement of more proteins. In addition, the findings of this study can only be extrapolated to populations with similar characteristics to participants of the InCHIANTI study and should be confirmed in other populations to ensure robustness of the results. We decided “a priori” not to adjust our analysis for specific diseases or multi‐morbidity because diseases may be on the causal pathway between biomarkers and mobility loss, and a mediation analysis focused on the identified biomarkers should be performed in future studies. Lastly, we cannot infer causality because of the epidemiological nature of this study.

### 3.1 Conclusions and future directions

The present study demonstrated that plasma CTSS, THBS2, and GDF15 provide information on the risk of future development of mobility disability in community‐dwelling adults. We also found other proteins nominally associated with mobility loss that overall point to cellular inflammation, phagocytosis, and perhaps also cellular senescence as major pathways involved in mobility loss with aging. Future investigations are needed to examine the roles of these proteins in the biological pathways leading to mobility disability.

## 4 EXPERIMENTAL PROCEDURES

### 4.1 Study population and design

This study uses longitudinal data from the InCHIANTI study, conducted in Tuscany, Italy. The rationale, design, and data collection have been described elsewhere (Ferrucci et al., 2000). In the present analysis, we selected women and men aged 60 and older who had plasma proteomics data and completed 400‐m walk test at fast pace at baseline visit and who had also concurrent data on BMI, education level, smoking status, physical activity, global cognitive assessment, and depression symptoms at baseline visit. A total of 660 InCHIANTI participants (60–94 years) were eligible for this study. Median follow‐up time was 8.57 years [interquartile, 3.20–9.08 years]. Participants received an extensive description of the study and participated after written informed consent. The participants were seen again for follow‐up visits at 3 years (2001–2003), 6 years (2004–2006), and 9 years (2007–2009) after enrollment at which time they underwent a repeated assessment of gait speed. The study protocol complied with the Declaration of Helsinki and was approved by the Italian National Institute of Research and Care on Aging Ethical Committee and by the Ethical Committee of the Florence ASL 10 regional health authority.

### 4.2 Measurement of plasma proteomics

Venous blood was obtained in the early morning after an overnight fast, immediately stored at 4°C, centrifuged within 4 hr, then immediately aliquoted, and frozen at −80°C. The collection of EDTA plasma in both studies was consistent with guidelines for protein biomarker work (Tuck et al., 2009). Extensive pre‐analytical studies of proteins for proteomic studies using LC‐MS/MS show that plasma proteins are stable for 14–17 years in storage at −80°C and for up to 25 freeze–thaw cycles (Hassis et al., 2015; Zimmerman, Li, Yarbrough, Slebos, & Liebler, 2012). Plasma proteomics was measured using the 1.3k HTS SOMAscan assay (SOMALogic, Boulder, CO), which has been described in detail elsewhere (Candia et al., 2017; Kraemer et al., 2011). The proteomics data were expressed as abundance in relative fluorescence units (RFU). Data normalization was conducted according to the Trans‐NIH Center for Human Immunology (CHI) pipeline. First, hybridization control normalization removes individual sample variance on the basis of signaling differences between microarray or Agilent scanner. Second, median signal normalization removes inter‐sample differences within a plate due to technical differences such as pipetting variation. Third, calibration normalization removes variance across assay runs. Additionally, interplate normalization procedures using CHI site‐specific calibrators from pooled healthy donors were performed to allow quality control of the normalization across all experiments conducted at the CHI (Candia et al., 2017). An interactive Shiny web tool was used during the CHI QC process (Cheung et al., 2017). The overall technical variability of the assay is low, with a median intraplate CV in the ~3% to 4% range. After removing 12 hybridization controls, 4 viral proteins (HPV type 16, HPV type 18, isolate BEN, and isolate LW123), and 5 SOMAmer reagents that were reported to be nonspecific (P05186; ALPL, P09871; C1S, Q14126; DSG2, Q93038; TNFRSF25, and Q9NQC3; RTN4), a total of 1,301 proteins were used in this analysis.

### 4.3 Mobility performance measure

We collected data on a 400‐m walk at fast pace. A. The examiners asked participants to walk as fast as possible without running for 10 laps in a 20‐m walking course that was set with two cones at each end (Simonsick et al., 2001). The time to complete 400 m was recorded. For the analysis, we used gait speed that was calculated by the distance divided by total time to complete the 400‐m walk (m/s). If the participant interrupted the test for a rest, the time count stopped and restarted when he/she started again. If the participant definitively abandoned the test, the time count was halted. Exclusion criteria for the 400‐m walk test were as follows: (a) Heart rate was <40 or >135 bpm, (b) anterior MI in the last 3 months, (c) cardiac surgical intervention in the last 3 months, (d) episodes of angina in the last 3 months, (e) severe dyspnea and dyspnea at rest in the last 3 months, (f) loss of consciousness in the last 3 months, (g) systolic BP >180 mm Hg or diastolic BP >100 mm Hg, (h) pathologic changes in ECG, (i) difficulty in keeping the feet in parallel for 10 s., and (j) difficulty in walking 8 m. In addition, criteria for termination of the test were as follows: (a) palpitations, (b) chest pain, constriction, and feeling of oppression, (c) respiratory difficulty of dyspnea, (d) sensation of fainting, empty head, or postural instability, (e) pain in the lower limbs, (f) vertigo, (g) muscle fatigue, and (h) refusal.

### 4.4 Incidence of mobility disability during a 9‐year follow‐up

All participants in this analysis completed the 400‐m walk at the baseline visit. Incident mobility disability was defined as follows: during the follow‐up period, if (a) the participant did not attempt the 400‐m walk test, (b) did not complete the 400‐m walk, or (c) met the exclusion criteria for the 400‐m walk or the criteria for termination described above. Time to develop mobility disability was calculated as the number of days between the performance measure at baseline and that of follow‐up visit that the participant first met the criteria of mobility disability. For those who did not develop mobility disability, the follow‐up time was censored at the date of 9‐year follow‐up visit. We treated those who died during the follow‐up period without developing mobility disability as censored at the day of their death and those who missed follow‐up without developing mobility disability as censored at the last follow‐up visit day. To obtain information on vital status, the study utilized data from the Mortality General Registry data operated by the Tuscany Region and death certificates deposited immediately after death at the Registry office in the municipality of residence.

### 4.5 Covariates

Socio‐demographic variables (sex, age, and education year), smoking status (current smoker or not), physical activity, cognitive performance, and depression symptoms were collected using standardized questionnaires. All participants were examined by a trained geriatrician. Weight was measured using a high‐precision mechanical scale. Standing height was measured to the nearest 0.1 cm. By using a self‐reported physical activity questionnaire to ask physical activity level in the last year, we defined as inactive if the participants reported “Hardly any physical activity,” or “Mostly sitting or some walking.” To evaluate cognitive performance and symptom of depression, we used the MMSE (0–30 points) and the CES‐D (0–60 points), respectively.

### 4.6 Statistical methods

Descriptive data are shown as the means ± *SD* for continuous variables and percentages for categorical variables. Age‐ and sex‐adjusted group comparisons in baseline variables were tested by linear regression models or logistic regression models to account for aging and sex effects on anthropometric and performance measures. All protein samples above/below 3 *SD* from mean values were excluded as outliers. Z‐score of each protein was used for the analysis.

To test whether plasma proteomics at baseline predict the time to develop mobility disability, we used a multivariable Cox proportional hazards model. The model was adjusted for sex, baseline age, baseline BMI, education level, baseline smoking status, baseline physical activity, baseline MMSE, baseline CED‐S, and baseline gait speed. Once we found significance, we additionally put all significant proteins into the model to test if these proteins independently predict incident mobility disability. Furthermore, to discover promising proteins, we performed a multivariable Cox proportional hazards model with backward selection method while including covariates. Additionally, we introduced interaction term between protein and sex to test whether the association differs by sex.

SAS software version 9.4 for Windows (SAS Institute, Inc., Cary, NC) was used for all data processing and statistical analyses. We set the level of statistical significance as *p* < .05 (two‐sided) to discover novel biomarkers and pathways and as false discovery rate q‐value less than 0.05 (two‐sided) to identify the strong biomarkers associated with incident mobility disability.

### 4.7 Pathway analysis and the senescence‐associated secretory phenotype (SASP)

For a better understanding of candidate proteins that were associated with the risk of developing mobility disability with a nominal *p*‐value <.05, we explored what pathways were enriched by using the Kyoto Encyclopedia of Genes and Genomes (KEGG) pathway enrichment in the Database for Annotation, Visualization and Integrated Discovery (DAVID, https://david.ncifcrf.gov/). In addition, we checked those proteins that have been reported as SASP proteins by using the SASP Atlas (Basisty et al., 2019).

## CONFLICT OF INTEREST

All authors declare no conflicts of interest.

## AUTHOR CONTRIBUTIONS

LF directed and supervised the project. SOMAscan assay was run by GF, and JC conducted proteomic data normalization and cleaning. YO, RDS, and LF configured the concept and design of study and manuscript preparation. YO and TT conducted the statistical analysis. YO, LF, TT, and RDS contributed to the interpretation of data. YO prepared the manuscript, and all authors have contributed to and approved the final version of the manuscript.

## Supporting information

Table S1

Tables S2‐S4

## Data Availability

Data proteomic data generated from this study are available upon request through submission of proposals at the InCHIANTI study Web site http://inchiantistudy.net/wp/
