# Supplementary material for: Plasma proteomic signature of the risk of developing mobility disability: A 9‐year follow‐up
Source: Aging Cell. 2020 Mar 10;19(4):e13132. doi: 10.1111/acel.13132 (PMC7189986; doi:10.1111/acel.13132)
Supplement: Supplementary file 2 — Tables S2‐S4 [file ACEL-19-e13132-s002.docx]

| **Supplemental Table 2.** Result of multivariate Cox proportional hazard risk model including significant proteins | | | | |
| --- | --- | --- | --- | --- |
|  |  |  |  |  |
| **Parameter** | **HR** | **95%CI** | | **p-value** |
| **CTSS** | 1.22 | 1.06 | 1.40 | 0.004 |
| **THBS2** | 1.22 | 1.00 | 1.48 | 0.046 |
| **GDF15** | 1.26 | 1.04 | 1.54 | 0.019 |
| Sex | 1.20 | 0.90 | 1.59 | 0.215 |
| Age | 1.04 | 1.01 | 1.06 | 0.006 |
| BMI | 1.01 | 0.98 | 1.04 | 0.542 |
| Education | 0.60 | 0.44 | 0.81 | 0.001 |
| Smoking | 0.86 | 0.60 | 1.24 | 0.415 |
| Inactive | 0.87 | 0.66 | 1.14 | 0.313 |
| Gait speed | 0.15 | 0.07 | 0.30 | <.0001 |
| CTSS, cathepsin S; GDF15, | | |  |  |
| growth/differentiation factor 15; | | | |  |
| THBS2, thrombospondin-2 | | |  |  |

| **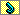Supplemental Table 3.** Result of Cox proportional hazard risk model, including covariates and significant 75 proteins | | | | |
| --- | --- | --- | --- | --- |
| **Parameter** | **HR** | **95%CI** | |  |
| Sex† | 1.13 | 0.72 | 1.75 | 0.598 |
| Age† | 1.02 | 0.98 | 1.06 | 0.331 |
| BMI† | 0.99 | 0.94 | 1.03 | 0.479 |
| Education† | 0.46 | 0.29 | 0.71 | 0.001 |
| Smoking† | 0.59 | 0.35 | 1.01 | 0.054 |
| Inactive† | 0.74 | 0.51 | 1.07 | 0.110 |
| Gait speed† | 0.13 | 0.05 | 0.35 | <.0001 |
| CTSS† | 1.01 | 0.82 | 1.23 | 0.965 |
| THBS2† | 1.49 | 1.09 | 2.03 | 0.012 |
| GDF15† | 1.37 | 1.04 | 1.81 | 0.027 |
| CD38 | 1.85 | 1.19 | 2.87 | 0.006 |
| MSTN | 3.55 | 1.72 | 7.34 | 0.001 |
| MMP3 | 0.61 | 0.37 | 0.99 | 0.047 |
| BCAM | 1.56 | 1.13 | 2.16 | 0.007 |
| CNTN1 | 0.50 | 0.36 | 0.68 | <.0001 |
| PGLYRP1 | 1.51 | 1.12 | 2.04 | 0.008 |
| GNS | 1.58 | 1.01 | 2.47 | 0.044 |
| NPPB | 0.59 | 0.39 | 0.90 | 0.015 |
| FGR | 2.88 | 1.80 | 4.63 | <.0001 |
| TPO | 3.40 | 1.80 | 6.44 | 0.0002 |
| SPON1 | 0.72 | 0.51 | 1.00 | 0.049 |
| SIGLEC1 | 0.44 | 0.21 | 0.93 | 0.031 |
| CHST15 | 1.39 | 1.08 | 1.80 | 0.012 |
| MMP13 | 3.35 | 1.80 | 6.26 | 0.0001 |
| MAPK13 | 1.60 | 1.13 | 2.27 | 0.008 |
| TNFSF14 | 2.15 | 1.13 | 4.10 | 0.020 |
| FTH1 FTL | 0.70 | 0.58 | 0.86 | 0.0004 |
| BCAM, Basal cell adhesion molecule; CD38, ADP-ribosyl cyclase/cyclic ADP-ribose hydrolase 1; CHST15, Carbohydrate sulfotransferase 15; CNTN1, Contactin-1; CTSS, Cathepsin S; FGR, Tyrosine-protein kinase Fgr; FTH1 FTL, Ferritin heavy chain; GDF15, Growth/differentiation factor 15; GNS, N-acetylglucosamine-6-sulfatase; MAPK13, Mitogen-activated protein kinase 13; MMP13, Collagenase 3; MMP3, Stromelysin-1; MSTN, rowth/differentiation factor 8/11; NPPB, Natriuretic peptides B; PGLYRP1, Peptidoglycan recognition protein 1; SIGLEC1, Sialoadhesin; SPON1, Spondin-1; THBS2, Thrombospondin-2; TNFSF14, Tumor necrosis factor ligand superfamily member 14; TPO, Thyroid peroxidase. †Forcefully included in a model with backward selection method | | | | |
|  |  |  |  |  |
|  |  |  |  |  |
|  |  |  |  |  |
|  |  |  |  |  |
|  |  |  |  |  |
|  |  |  |  |  |

**Supplementary Table 4.** The Senescence-Associated Secretory Phenotype (SASP) in our notable proteins.


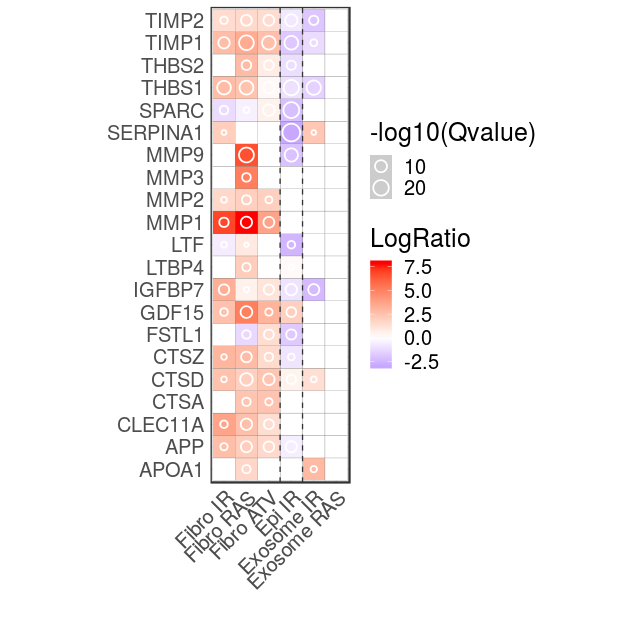


We used the SASP Atlas (Basisty et al., 2019).

Basisty, N., Kale, A., Jeon, O., Kuehnemann, C., Payne, T., Rao, C., . . . Schilling, B. (2019). A Proteomic Atlas of Senescence-Associated Secretomes for Aging Biomarker Development. 604306. doi:10.1101/604306 %J bioRxiv
